# Sexual Dysfunctions in Patients Receiving Opioid Agonist Treatment and Heroin-Assisted Treatment Compared to Patients in Private Practice—Identifying Group Differences and Predictors

**DOI:** 10.3389/fpsyt.2022.846834

**Published:** 2022-03-22

**Authors:** Maximilian Meyer, Patrick Brunner, Leonie Geissmann, Martin Gürtler, Fabienne Schwager, Rowena Waldis, Marc Vogel, Gerhard A. Wiesbeck, Kenneth M. Dürsteler

**Affiliations:** ^1^Clinic for Adult Psychiatry, University Psychiatric Clinics, University of Basel, Basel, Switzerland; ^2^Division of Cognitive Neuroscience, Department of Psychology, University of Basel, Basel, Switzerland; ^3^Health Center Allschwil (Gesundheitszentrum Allschwil AG), Allschwil, Switzerland; ^4^Department of Clinical Research, University of Basel, Basel, Switzerland; ^5^Department for Psychiatry, Psychotherapy and Psychosomatics, Psychiatric Hospital, University of Zurich, Zurich, Switzerland

**Keywords:** opioid use disorder, sexual dysfunction, erectile dysfunction, opioid dependence, heroin dependence

## Abstract

**Background and Aims:**

Sexual dysfunctions (SDs) show a marked impact on a person’s general wellbeing. Several risk-factors like physical and mental illnesses as well as alcohol and tobacco use have to date been identified to contribute to the occurrence of SDs. The impact of opioid-agonist treatment (OAT) on SDs remains unclear, with some studies demonstrating an improvement after methadone maintenance treatment (MMT) initiation. However, no studies on the prevalence and predictors of SDs in heroin-assisted treatment (HAT) exist to date.

**Methods:**

A cross-sectional study was conducted with patients from a MMT center (*n* = 57) and a center specializing in HAT (*n* = 47). A control group of patients with mild transient illnesses (*n* = 67) was recruited from a general practitioner (GP). The International Index of Erectile Function, the Female Sexual Function Index, as well as measurements for psychological distress, depressive state, nicotine dependence, and high-risk alcohol use were employed. Patients also completed a self-designed questionnaire on help-seeking behavior regarding sexual health. Mann-Whitney-U tests and chi-square tests were performed for group comparisons and binary logistic regression models were calculated.

**Results:**

Twenty-five percent of the GP sample (*n* = 17), 70.2% (*n* = 40) of the MMT sample, and 57.4% (*n* = 27) of the HAT sample suffered from SDs at the time of study conduction. OAT patients differed significantly from GP patients in depressive state, high-risk alcohol use, nicotine dependence, and psychological distress. Age, depressive state, and opioid dependence predicted the occurrence of SDs in the total sample. No differences between OAT and GP patients were found regarding help-seeking behavior.

**Discussion:**

Age, depressive state, and opioid dependence predicted the occurrence of SDs in the total sample. It remains unclear whether SDs are caused by opioid intake itself or result from other substance-use related lifestyle factors, that were not controlled for in this study. A lack of help-seeking behavior was observed in our sample, underlining the importance of clinicians proactively inquiring about the sexual health of their patients.

**Conclusion:**

The high prevalence of SDs observed in MMT does not differ from the prevalence in HAT. Clinicians should actively inquire about their patients’ sexual health in GP and OAT centers alike.

## Introduction

Sexual dysfunctions (SDs) have a marked impact on the psychosocial wellbeing of affected individuals and are associated with anxiety and depression (1), poorer quality of life (2), diminished confidence, and low self-esteem (3). SDs also have detrimental effects on the relationship quality and the partners wellbeing (4). In women, SDs can be subsumed to difficulties in sexual desire, arousal, and pain (5). In men, the most common SDs include erectile dysfunction (ED) and premature ejaculation (6). Reported prevalence rates of SDs in the general population are inconsistent and vary with assessment technique, definition being used, underlying comorbidities and age (7, 8). Scholars have found the prevalence of male SDs to be lower than female SDs, stating estimations between 10 and 52% for men and between 12 and 80% for women (9, 10). In premenopausal women, recent estimations range from 41% (11) to 51% (12). The prevalence of ED is estimated at 16% across the age of 20–75 years (13), whereas the prevalence of premature ejaculation varies from 8 to 30% for all age groups (6).

Notably, prevalence estimates show a great variance and are further complicated by the fact that in the health care system SDs are commonly not inquired for Brookmeyer et al. (14). In primary care, only 10–16% of general practitioners actively ask for sexual concerns (15) and the rate of sexual history taking in urologists and gynecologists was found to be 23 and 8%, respectively (16, 17). Additionally, only a small fracture of individuals affected by SDs seek professional help for sexual concerns (18).

Several risk factors for SDs have been identified. Somatic illnesses and psychiatric disorders have been found to negatively impact sexual functioning, whereas findings on lifestyle factors such as alcohol consumption and tobacco smoking remain partly inconclusive for male and female SDs alike (19). The use of opioids and opioid use disorders are also associated with impaired sexual functioning (20). Due to a substance-using lifestyle, chronic heroin users usually exhibit a multitude of risk factors for sexual dysfunctions (e.g., poor nutrition, smoking, alcohol use, mental comorbidity). The clinical gold standard in treatment of these patients is opioid agonist treatment (OAT), in which an alternative opioid, such as methadone, buprenorphine, slow-release oral morphine, or pharmaceutical diacetylmorphine (heroin, DAM) is prescribed. In many countries, methadone maintenance treatment (MMT) is currently the most common form of OAT (21) and only few countries have permitted and introduced heroin-assisted treatment (HAT) (22). As in the general population, reported prevalence rates of SDs in individuals on MMT vary with some scholars reporting rates as low as 14% (23) up to 93% for men (24) and 56.6% for women (25). However, the lack of help-seeking behavior in regard to SDs is present in MMT as well, making precise prevalence estimates difficult: one study reported that in their sample only 8% of men with ED consulted a physician (26). To our knowledge, no prevalence rates for SDs in individuals receiving HAT or non-treatment-seeking illicit heroin users exist, although data from one retrospective study indicates improvement of SD after MMT initiation (27).

Indeed, evidence suggests that OAT may have a positive impact on hormonal levels and sexual functioning. It is well-known that heroin exposure can impair hypothalamic-pituitary-gonadal function, resulting in irregular menses and secondary amenorrhea (28). Early studies found prevalence rates of menstrual disorders among heroin-dependent women of 85–90% (29, 30). Cycle-length irregularity and amenorrhea were also common among 133 women in MMT, but each additional week on MMT was associated with decreased risk of both short and long cycles, and 16 of the 27 women who were amenorrhoeic at study entry restarted menses in that study (31). In men, it has been reported that MMT improves SDs after 6 months of treatment (32). However, this was recently contradicted by a study that found similar rates of hypogonadism in MMT and heavy heroin use (33). Additionally, differences on the extent to which OAT affects sexual functioning have been found. It has been repeatedly demonstrated that SDs occur less frequently in OAT with buprenorphine compared to MMT (34–36).

In summary, the impact of OAT on sexual functioning remains controversial, with a noticeable lack of literature on SDs in HAT. Studies found high prevalence rates of SDs in individuals receiving OAT without it being certain whether the opioid intake itself causes these symptoms. To this day, the extent to which comorbidities and other factors associated with drug-related lifestyle contribute to the occurrence of SDs is unclear. Also, there are no studies available on prevalence rates for SDs in individuals receiving HAT and contributing factors have to date not been identified.

### Aim

The aim of the present study was to assess sexual functioning in opioid-dependent patients on OAT with either MMT or HAT (injectable diacetylmorphine) as compared to a control sample of general practitioner patients with mild transient illnesses. Three research questions were formulated in line with this aim: 1. Do patients receiving OAT differ from general practitioner patients with mild transient illnesses with respect to help seeking behavior, the prevalence, and the extent of sexual dysfunctions? 2. Do patients receiving MMT differ from patients receiving HAT in this regard? 3. What factors do contribute to the occurrence of sexual dysfunctions?

## Materials and Methods

### Study Design and Setting

The study was conducted in a cross-sectional design at a general practitioner (GP, private practice) and at two opioid maintenance centers of the Psychiatric University Clinics of Basel, Switzerland from March 18 to 31, 2012. One center provided MMT (mostly methadone and slow-release oral morphine). The other center specialized in HAT (injectable diacetylmorphine alone or in combination with other opioid-agonists including oral diacetylmorphine). In both centers, opioid medications were tailored to the needs and the substance-use history of the individual patient and were provided as part of a comprehensive treatment program covering a broad range of therapeutic elements and supportive services.

### Study Sample

Eligible for participation were either patients who had an appointment with their GP due to a mild transient illness and all patients receiving either MMT or HAT in the above-mentioned centers and who did not show evidence of intoxication during recruitment. OAT patients were recruited face-to-face at each clinic while waiting in line for dispensing and GP patients were recruited during their visit at the private practice.

Inclusion criteria for opioid-dependent patients were age between 18 and 65 years, presence of sexual activity (including masturbation) and a sexual relationship (including contacts with sex workers) within the past 4 weeks, and ability to understand and communicate in German to complete the study measurements. Exclusion criteria were current treatment with antiviral medication for viral hepatitis or HIV, androgen replacement therapy, or phosphodiesterase type 5 inhibitors, medication or other medical conditions associated with impaired sexual functioning, and participation in OAT for less than 4 weeks.

Inclusion criteria for GP-patients were age of 18 years or older and ability to understand and communicate in German to complete the study measurements. Exclusion criteria were the presence of a somatic illness, medication or other medical condition that is associated with impaired sexual functioning as well as age over 65 years. A separate study on the GP sample alone has been published previously by the study team (37).

In total, 171 patients participated in the study, 67 of whom were recruited in private practice. Fifty-seven were recruited from the MMT center and 47 patients from the HAT center. The sample size was derived from comparable studies in the field (21, 38). All patients and healthy controls were of Caucasian ancestry.

### Measures and Data Collection

Demographic data concerning age, sex, nationality, highest education level achieved, employment/social support status, civil status, and housing situation were reported by each participant.

#### Participant-Rated Measurements

All patients completed a battery of standardized self-report instruments in paper-pencil form. While completing the questionnaires participants had the opportunity to ask the investigator assessing the data if they had any further queries.

International Index of Erectile Function (IIEF): The IIEF (39) is a validated 15-item self-administered questionnaire assessing different domains of sexual functioning in men (erectile function, orgasmic function, sexual desire, intercourse and overall satisfaction). The optimal cut-off for the total score has been found to be 53 (40). It has also been validated in German (40), and the original five-factor structure could be confirmed (41).

Female Sexual Function Index (FSFI): The FSFI (42) is a 19-item validated self-report questionnaire assessing the key dimensions of sexual functioning in women over the past 4 weeks (desire, arousal, lubrication, orgasm, satisfaction, and pain). A cut-off score of 26.55 has been found to show the best sensitivity (0.733) und specificity (0.889) for differentiating women with and without SD, with higher scores indicating better sexual functioning (43).

Center of Epidemiologic Studies—Depression Scale (CES-D): Depressive symptoms were assessed with the German adaption of the CES-Depression Scale (Allgemeine Depressionsskala-Lang, ADS-L) (44, 45). The ADS-L is a 20 item self-report measure of depressive mood. Respondents rate items on a 4-point Likert-type scale from 0 (never/very rarely) to 4 (always/nearly all the time). The scale is widely used and reliability and validity data have been documented for the use within general and clinical populations (46).

Symptom Checklist-27 (SCL-27): The SCL-27 (47) is a modification of the German Symptom Checklist-90-R (48) and evaluates psychological distress caused by physical disorders or complaints on six subscales. Each subscale consists of 4–6 items and each item is rated on a 5-point Likert scale. The Global Severity Index (GSI) is calculated from the SCL and provides a global composite score. The positive predictive value was 0.91 at the cut-off score of 0.5 for discriminating psychiatric patients from a reference sample (49).

Alcohol Use Disorders Identification Test-Consumption (AUDIT-C): This 3-item screening questionnaire developed by the World Health Organisation to identify harmful or hazardous alcohol consumption. Its utility as a screening instrument for hazardous drinking has also been shown in substance-dependent patients, with a sensitivity of 0.97, a specificity of 0.69 and a positive predictive value of 0.65 (50, 51). AUDIT-C items are scored on a scale of 0–12 and responses to each of the 3 items are assigned 0–4 points. The recommended AUDIT-C threshold for unhealthy alcohol use differs between men (≥ 4) and women (≥3) (52).

Fagerström-Test for Nicotine Dependence (FTND-G): The FTND (53) was designed to provide an ordinal measure of nicotine dependence related to cigarette smoking. It contains six items that evaluate the quantity of cigarette consumption, the compulsion to use, and dependence. Yes/no items are scored from 0 to 1 and multiple-choice items are scored from 0 to 3. The items are summed to yield a total score of 0 to 10. The internal consistency of FTND is moderate (Cronbach’s alpha = 0.56) (54).

A self-designed questionnaire was employed to inquire about the role of sexual health during patients’ contact with health care providers to assess help-seeking behavior. Items were rated on a 5-point Likert scale or in “yes”/”no”-form.

### Statistical Analysis

Statistical analysis was conducted with SPSS version 28 (IBM). As the data were not normally distributed Mann-Whitney-U tests were performed to calculate differences in scale scores between groups. Chi-square tests were used to determine differences in prevalence rates and help-seeking behavior. Binary logistic regression models were calculated to identify variables predictive of SD. Included variables were tested for multicollinearity by calculating the variance inflation factor. Level of significance was set at *p* < 0.05 for all calculations. Missing data was replaced by the median of the respective variable when it became necessary.

### Ethics

All participants gave written informed consent after being informed about the aims and procedures of the study in detail. Participants did not receive any compensation for participation in the study. The study protocol was approved by the local ethics committee (Ref. Nr. EK 31/11) and was conducted in accordance with the Declaration of Helsinki.

## Results

Table 1 provides the sociodemographic characteristics of the sample.

**TABLE 1:** Sample characteristics.

		GP (*n* = 67)	MMT (*n* = 57)	HAT (*n* = 47)	Total sample (*n* = 171)
Age		*M* = 49.8 (*SD* = 12.0)	*M* = 40.8 (*SD* = 8.9)	*M* = 43.7 (*SD* = 6.5)	*M* = 41.6 (*SD* = 9.8)
Sex	Male	*n* = 37 (55.2%)	*n* = 36 (63.2%)	*n* = 29 (61.7%)	*n* = 102 (59.6%)
	Female	*n* = 30 (44.8%)	*n* = 21 (36.8%)	*n* = 18 (38.3%)	*n* = 69 (40.4%)
Professional degree		*n* = 62 (92.5%)	*n* = 39 (68.4%)	*n* = 28 (59.6%)	*n* = 129 (75.4%)
Housing situation	Alone	*n* = 14 (20.9%)	*n* = 35 (61.4%)	*n* = 28 (59.6%)	*n* = 77 (45%)
	With partner	*n* = 48 (71.6%)	*n* = 9 (15.8%)	*n* = 6 (12.8%)	*n* = 63 (36.8%)
	With parents	*n* = 4 (6.0%)	*n* = 5 (8.8%)	*n* = 5 (10.6%)	*n* = 14 (8.2%)
	Assisted living	-	*n* = 4 (7.0%)	*n* = 4 (8.5%)	*n* = 8 (4.7%)
	Shared apartment	*n* = 1 (1.5%)	*n* = 3 (5.3%)	*n* = 2 (4.3%)	*n* = 6 (3.5%)
	Not specified (missing)	-	*n* = 1 (1.8%)	*n* = 2 (4.3%)	*n* = 3 (1.8%)
Civil status	Unmarried	*n* = 25 (37.3%)	*n* = 47 (82.5%)	*n* = 36 (76.6%)	*n* = 108 (63.2%)
	Married	*n* = 36 (53.7%)	*n* = 2 (3.5%)	-	*n* = 38 (22.2%)
	Divorced	*n* = 5 (7.5%)	*n* = 5 (8.8%)	*n* = 8 (17.0%)	*n* = 18 (10.5%)
	Separated	*n* = 1 (1.5%)	*n* = 1 (1.8%)	*n* = 1 (2.1%)	*n* = 3 (1.8%)
	Widowed	-	*n* = 1 (1.8%)	-	*n* = 1 (0.6%)
	Not specified (missing)	-	*n* = 1 (1.8%)	*n* = 2 (4.3%)	*n* = 3 (1.8%)
One or more children		*n* = 34 (50.7%)	*n* = 23 (40.4%)	*n* = 14 (29.8%)	*n* = 71 (41.5%)
Alcohol use disorder in family		*n* = 12 (17.9%)	*n* = 20 (35.1%)	*n* = 12 (25.5%)	*n* = 44 (25.7%)
Any other SUD in family		*n* = 6 (9.0%)	*n* = 7 (12.3%)	*n* = 8 (17.0%)	*n* = 21 (12.3%)

*GP, general practitioner; MMT, methadone maintenance treatment; HAT, heroin-assisted treatment; SUD, substance use disorder.*

### Prevalence of Sexual Dysfunction in the Sample

Occurrence of sexual dysfunction was defined as IIEF score lower than 53 for males (40) and an FSFI score lower than 26.55 for females (43). Prevalence of SDs in GP patients 25.4% (*n* = 17) differed significantly from the prevalence found in OAT patients 64.4% (*n* = 67) as determined by the chi-square test [χ^2^(1, *n* = 171) = 24.9, *p* < 0.001]. No statistically significant difference was found between prevalence in HAT patients (57.4%, *n* = 27) and prevalence in MMT patients (70.2%, *n* = 40) [χ^2^(1, *n* = 104) = 1.8, *p* = 0.18].

When comparing male to female patients, no differences were found in the total sample [χ^2^(1, *n* = 171) = 1.6, *p* = 0.20], the GP patients [χ^2^(1, *n* = 67) = 0.6, *p* = 0.43], all OAT patients [χ^2^(1, *n* = 104) = 2.7, *p* = 0.10], and the HAT patients [χ^2^(1, *n* = 47) = 0.0, *p* = 0.84]. In MMT patients, SDs occurred significantly more often in male patients [χ^2^(1, *n* = 57) = 6.5, *p* = 0.01].

### Differences in the Extent of Sexual Dysfunction Between Patients Receiving Opioid-Agonist Treatment and General Practitioner Patients With Mild Transient Illnesses

Descriptive statistics and differences as determined by the Mann-Whitney-U test of FSFI and IIEF scores and their subdomains are provided in Table 2. There was a significant difference in the total IIEF (*U* = 665.0, *p* < 0.001, *r* = 0.37) and FSFI (*U* = 155.5, *p* < 0.001, *r* = 0.63) scores between the GP patients and OAT patients, with GP patients showing significantly higher IIEF and FSFI scores.

**TABLE 2:** Sexual functioning in GP and OAT patients.

		GP (*n* = 30)	OAT (*n* = 39)	*p*
FSFI full scale score		*M* = 28.27 (*SD* = 6.72)	*M* = 15.28 (*SD* = 9.95)	<0.001
FSFI domains	Desire	*M* = 3.68 (*SD* = 1.14)	*M* = 2.55 (*SD* = 1.44)	<0.001
	Arousal	*M* = 4.52 (*SD* = 1.38)	*M* = 2.39 (*SD* = 2.03)	<0.001
	Lubrication	*M* = 5.02 (*SD* = 1.57)	*M* = 2.46 (*SD* = 2.23)	<0.001
	Orgasm	*M* = 5.05 (*SD* = 1.40)	*M* = 2.06 (*SD* = 2.01)	<0.001
	Satisfaction	*M* = 5.07 (*SD* = 1.04)	*M* = 2.90 (*SD* = 1.55)	<0.001
	Pain	*M* = 4.93 (*SD* = 1.60)	*M* = 2.90 (*SD* = 2.57)	<0.001
		GP (*n* = 37)	OAT (*n* = 65)	
IIEF full scale score		*M* = 57.73 (*SD* = 17.20)	*M* = 40.80 (*SD* = 22.51)	<0.001
IIEF domains	Erectile function	*M* = 24.70 (*SD* = 8.15)	*M* = 16.51 (*SD* = 11.14)	<0.001
	Orgasmic function	*M* = 8.89 (*SD* = 2.95)	*M* = 5.68 (*SD* = 4.43)	<0.001
	Sexual desire	*M* = 6.95 (*SD* = 1.61)	*M* = 6.25 (*SD* = 2.35)	0.192
	Intercourse satisfaction	*M* = 9.49 (*SD* = 5.01)	*M* = 5.02 (*SD* = 5.78)	<0.001
	Overall satisfaction	*M* = 7.70 (*SD* = 2.26)	*M* = 7.35 (*SD* = 2.18)	0.355

*Mann-Whitney-U tests were performed to compare groups.*

*FSFI, Female Sexual Function Index; IIEF, International Index of Erectile Function; GP, general practitioner (sample); OAT, opioid-agonist treatment (sample); M, mean; SD, standard deviation.*

In a next step, only patients who met the respective IIEF- and FSFI-cut-offs were included in the calculation of the Mann-Whitney-U test to examine whether patients differed in the severity of SD. No differences were found between GP and OAT patients with SD in IIEF total and subdomain scores. In female patients, we found the total FSFI score as well as the orgasm and satisfaction-domain scores to be significantly lower in OAT patients (*p* = 0.007, *p* = 0.007, and *p* < 0.001, respectively).

Groups also differed significantly in risk drinking behavior as determined by the AUDIT-C, psychological distress as determined by the SCL-27, severity of nicotine dependence as determined by the FTND, and depressive symptoms as determined by the ADS-L (Table 3).

**TABLE 3:** Descriptive statistics and differences in total scale scores as determined by Mann-Whitney-U tests.

Scale	GP (*n* = 67)	OAT (*n* = 104)	*p*
AUDIT-C	*M* = 3.04 (*SD* = 1.55)	*M* = 2.99 (*SD* = 3.26)	0.049
GSI	*M* = 0.29 (*SD* = 0.32)	*M* = 0.97 (*SD* = 0.81)	<0.001
FTND	*M* = 0.54 (*SD* = 1.46)	*M* = 4.92 (*SD* = 2.69)	<0.001
ADS-L	*M* = 10.75 (*SD* = 8.83)	*M* = 19.08 (*SD* = 9.85)	<0.001

*AUDIT-C, Alcohol Use Disorders Identification Test-Consumption; GSI, Global Severity Index; FTND, Fagerström-Test for Nicotine Dependence; ADS-L, Allgemeine Depressionsskala-Lang; M, mean; SD, standard deviation.*

### Help-Seeking Behavior Regarding Sexual Dysfunctions

**FIGURE 1:**
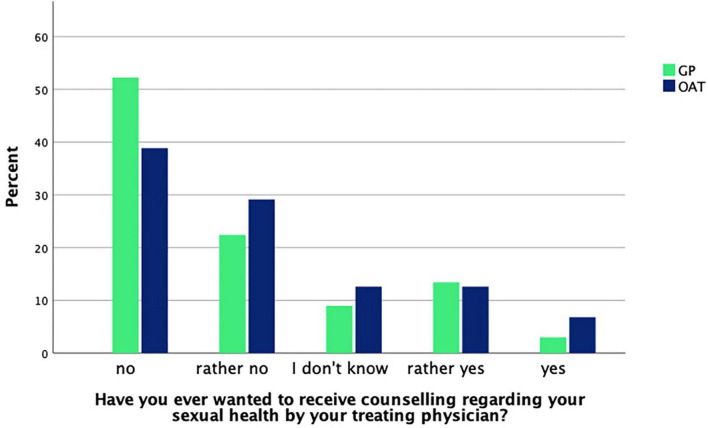
GP (*n* = 67) and OAT (*n* = 103) patients’ answers about whether they ever felt the need for counseling.

Patients were asked whether they had ever talked about their sexual health with their treating physician. About one third of OAT patients [29.1% (*n* = 30)] and GP patients [32.8% (*n* = 22)] answered to have done so. No significant difference was found between groups as determined by a chi-square test [χ^2^(1, *n* = 170) = 0.3, *p* = 0.61]. When asked whether their treating physician had ever inquired about their sexual health, 23.3% (*n* = 24) of OAT patients and 17.9% (*n* = 12) of GP patients responded “yes.” Again, there was no significant difference between groups [χ^2^(1, *n* = 170) = 0.7, *p* = 0.40]. The question whether patients ever wanted to receive counseling regarding their sexual health from their treating physician was also asked and responses are shown in Figure 1.

### Differences in the Extent of Sexual Dysfunction Between Patients Receiving Methadone Maintenance Treatment and Patients Receiving Heroin-Assisted Treatment

No significant difference in IIEF (*U* = 505.5, *p* = 0.828) and FSFI (*U* = 164, *p* = 0.481) total scores was found when comparing MMT to HAT patients. Additionally, there was no significant difference between groups in IIEF or FSFI subscales. Comparisons remained non-significant for total scores and subdomain scores after including only MMT and HAT patients who met the cut-offs of the IIEF and FSFI.

### Factors Contributing to the Occurrence of Sexual Dysfunction

Explorative binary logistic regression was performed to identify predictors of SD in our study population. Occurrence of sexual dysfunction was again defined as IIEF score lower than 53 or FSFI score lower than 26.55. Variables included were age, sex, whether patients suffered from opioid dependence (i.e., patients from the MMT and HAT center), depressive state as determined by the ADS-L, psychological distress as determined by the GSI, high-risk alcohol use as determined by the AUDIT-C and severity of nicotine dependence as determined by the FTND. The results are provided in Table 4.

**TABLE 4:** Logistic regression on sexual dysfunction prevalence in the total sample (*n* = 171).

Variables	*B*	*p*	OR (CI 95%)
Age	0.044	0.019	1.045 (1.007–1.084)
Sex	0.381	0.316	1.475 (0.690–3.152)
Depressive state	0.054	0.032	1.055 (1.005–1.109)
Nicotine dependence	–0.003	0.968	0.997 (0.853–1.165)
Psychological distress	0.146	0.701	1.158 (0.549–2.442)
High-risk alcohol use	–0.012	0.856	0.988 (0.863–1.130)
Opioid dependence	1.288	0.010	3.625 (1.369–9.595)

*Nagelkerkes R^2^ = 0.291; Hosmer-Lemeshow-test: χ^2^ = 2.211, p = 0.974.*

*OR, odds ratio; CI, confidence interval.*

To find out, whether predictive variables differed in the MMT and the HAT population, calculation was repeated for both patient groups, respectively. The opioid dependence variable was not included in these models. Whereas age remained significant in the MMT population (*B* = 0.086, *p* = 0.033, OR = 1.090), no variable was found to be predictive in the HAT sample.

## Discussion

This is the first study reporting on the prevalence of SDs and its contributing factors in HAT. We found the prevalence of SDs in our sample of GP, MMT and HAT patients to be 25, 70, and 57%, respectively. The prevalence of SDs observed in our sample of MMT patients is in line with previously reported findings (24, 25). Importantly, we did not find a significant difference in the respective MMT and HAT prevalence rates and our results therefore indicate that SDs are equally common in HAT patients. We also found that the severity of SDs as measured by the IIEF and the FSFI in HAT and MMT patients did not differ.

Unsurprisingly, we found SDs to be significantly more frequent in OAT patients when compared to GP patients with mild and transient illnesses. However, only female OAT patients experienced more severe SDs as indicated by lower total scale scores. This difference was not observed in male GP and OAT patients. GP patients also differed significantly from OAT patients in psychological distress, nicotine dependence, high-risk drinking behavior, and depressive symptoms. However, in our regression analysis, only depressive state emerged to be predictive for the occurrence of SDs in the total sample. In addition to depressive state, age and opioid dependence predicted SDs, which was also found in previous studies with patients receiving either methadone or buprenorphine (34).

Importantly, no difference between GP and OAT patients was observed regarding the lack of help-seeking behavior. Due to the high prevalence of SDs in individuals with opioid use disorder, clinicians should proactively inquire about their occurrence in these patients. However, in our sample, only 23% of OAT patients stated to have previously been asked about their sexual health by their treating physician. While the reasons for this finding can only be speculated on, low rates of sexual health examinations have previously been reported in the literature (15–17), underlining the need to further raise awareness of SDs in OAT.

No variable (including age) was found to predict SDs in the regression model only including HAT patients. This indicates that not all contributing factors were directly assessed by our study. It also raises the question whether substance-use related lifestyle factors or opioid agonists themselves play a large role in the emergence of SDs in this patient population. Regarding the latter, studies have repeatedly demonstrated that male patients in MMT suffer from SDs more often when compared to patients receiving treatment with buprenorphine (35, 36). This suggests that methadone itself contributes partly to the occurrence of SDs in opioid-dependent men. Since we found no difference in the prevalence of SDs between HAT and MMT patients, this might also hold true for DAM.

It remains unclear whether SDs are caused by opioid intake itself or result from other substance-use related lifestyle factors, that we did not control for. On a behavioral level, the substance-use related lifestyle frequently found in opioid-dependent patients has been suggested to affect various aspects of physical and mental health (38), which makes it hard to distinguish direct consequences of OAT from opioid-dependence-related factors. However, some factors that may be related directly or indirectly to opioid-dependence like tobacco smoking, psychological distress and higher rates of depression did not contribute to the occurrence of SDs in our sample. Other factors we did not specifically account for in our analysis might include mental comorbidities, disadvantageous health behaviors (e.g., illicit drug use), somatic illnesses, co-medication, and low socioeconomic status. In male MMT and buprenorphine patients, medical status, psychiatric illness, other current substance use, and civil status have previously been identified to be associated with SDs (55).

Studies comparing methadone and buprenorphine with regard to the occurrence of SDs in male and female patients have to date shown mixed results. Ruíz Ruíz et al. found the prevalence of SDs to be higher in the methadone group (36), whereas other studies did not find beneficial effects of buprenorphine (56). Therefore, the recommendation of switching from methadone to buprenorphine in MMT patients with SDs is not unequivocally supported by literature. Additionally, HAT is provided for individuals who did previously not respond to other forms of OAT, which most of the time precludes a change in their treatment regimen. Nonetheless, our data shows that just as in MMT, screening for SDs is equally important in HAT.

### Limitations

In addition to the above-mentioned shortcomings, the present study shows several other limitations. The first shortcomings concern the convenience sampling and the moderate sample size, which limit the generalizability of the present findings. Additionally, we did not control for daily opioid dosages, for co-morbidities and for illicit substance use, which may have a great impact on SDs. Also, we did not assess biological markers such as sexual hormone levels and the Body Mass Index. Finally, we did not analyze variables of interest for female and male patients individually.

## Conclusion

Patients in OAT suffer from SDs more often than GP patients with mild transient illness. Clinicians working in OAT centers and GP alike need to actively inquire about the sexual health of their patients. Depressive state, age, and opioid dependence predicted the occurrence of SDs in our sample of GP, MMT and HAT patients. No differences in frequency and extent of SDs were observed when comparing patients receiving HAT to MMT patients. Future studies are needed to assess whether the intake of DAM itself contributes to the occurrence of SDs.

## Data Availability Statement

The raw data supporting the conclusions of this article will be made available by the authors, without undue reservation.

## Ethics Statement

The studies involving human participants were reviewed and approved by Ethikkommission beider Basel (Ref. Nr. EK 31/11). The patients/participants provided their written informed consent to participate in this study.

## Author Contributions

PB, GW, and KD designed the study and wrote the study protocol. PB, MG, FS, MV, and KD were responsible for data collection and data management. MM, LG, RW, and KD conducted the data analyses and interpretation for the manuscript. MM, LG, and KD drafted the manuscript. All authors provided critical revision of the manuscript for important intellectual content.

## Conflict of Interest

The authors declare that the research was conducted in the absence of any commercial or financial relationships that could be construed as a potential conflict of interest.

## Publisher’s Note

All claims expressed in this article are solely those of the authors and do not necessarily represent those of their affiliated organizations, or those of the publisher, the editors and the reviewers. Any product that may be evaluated in this article, or claim that may be made by its manufacturer, is not guaranteed or endorsed by the publisher.
